# Adaptation of mtDNA content to endurance training, a cross-sectional study and an endurance training intervention

**DOI:** 10.1007/s00421-025-06016-5

**Published:** 2025-12-05

**Authors:** Isabel María Sánchez Lorente, Thomas Yvert, Tamara Iturriaga, Lara Sanchez-Barroso, Mar Larrosa, Margarita Pérez-Ruiz, Catalina Santiago-Dorrego

**Affiliations:** 1 ESBIDA Research Group, Department of Sport Sciences, Faculty of Medicine, Health and Sports, Universidad Europea de Madrid, Madrid, Spain; 2 ImFINE Research Group, Department of Health and Human Performance, Universidad Politécnica de Madrid, Madrid, Spain; 3 Department of Nursing, Facultad de Enfermería, Fisioterapia y Podología, Universidad Complutense de Madrid, Madrid, Spain; 4 InveCuid, Instituto de Investigación Sanitaria Hospital 12 de Octubre (imas12), Madrid, Spain; 5 Department of Nutrition and Food Science, Faculty of Pharmacy, Universidad Complutense de Madrid, Madrid, Spain; 6 ESBIDA Research Group, Department of Rehabilitation, Faculty of Medicine, Health and Sports, Universidad Europea de Madrid, Madrid, Spain

**Keywords:** Long HIIT, Running, Treadmill training, mtDNA copy number, Mitochondrial biogenesis

## Abstract

**Background:**

Maximal oxygen uptake (VO_2max_) is the gold standard for assessing cardiopulmonary fitness. However, the link between VO_2max_ and mitochondrial function is complex, with no direct causality proven. Analysing mtDNA copy number offers an innovative way to understand aerobic performance by measuring mitochondrial biogenesis, although its relation to physical performance and fitness is not well studied, especially in trained individuals.

**Objective:**

This study aimed to compare VO_2max_ and mtDNA copy number in blood leukocytes between highly trained runners and nontrained men. It also examined how leukocyte mtDNA content adapts to a 6-week HIIT aerobic training programme in nontrained individuals.

**Methods:**

We conducted a cross-sectional study with 20 highly trained runners and 20 nontrained healthy subjects. Then, we implemented a 6-week HIIT training programme for the nontrained group, comparing their VO_2max_ and mtDNA copy number to a control group. Participants trained 4 min/4 min HIIT treadmill running, three times a week.

**Results:**

The cross-sectional part showed that highly trained runners had significantly higher mtDNA copy numbers than nontrained subjects (*p* = 0.046; *d* = 0.652; 95% CI: [0.01, 1.28]; [medium–large effect]); further, we observed a significant positive correlation between mtDNA copy number and VO_2max_ combining both groups (*p* = 0.013; R^2^ = 0.153 [small–moderate effect]). After the 6-week HIIT programme, the intervention group showed significant increases in both VO_2max_ (*p* < 0.001) and mtDNA copy number (*p* = 0.015), with large and medium effect sizes, respectively. The intervention group’s mtDNA copy number increased by 321.6 ± 391.6%; 95% CI: [120.2, 522.9], compared to 12.8 ± 32.8%; 95% CI: [− 5.4, 30.9] in controls, indicating substantial interindividual variability, which could be attributed to a combination of biological factors, but we also observed indications of a potential responder versus non-responder pattern.

**Conclusion:**

These findings align with previous research, indicating that mtDNA copy number correlates with VO_2max_, being higher in highly trained runners than in nontrained subjects, and increasing with training after a 6-week HIIT intervention. This study is novel in showing that a 6-week HIIT programme significantly boosts leukocyte mtDNA content, enhancing our understanding of individual adaptations to endurance training.

## Introduction

Cardiorespiratory fitness represents the ability of the organism to transport oxygen and nutrients from the external environment to the mitochondria to transform them into energy, in the form of adenosine triphosphate (ATP), in order to perform physical activity. Since it is directly related to the integrated function of numerous systems, cardiorespiratory fitness is considered a reflection of total body health. In fact, it depends on pulmonary ventilation and gas diffusion to the blood, right and left ventricular function, ventricular–arterial coupling, efficient transport of blood from the heart to muscles to precisely meet their oxygen needs, and the ability of muscle cell mitochondria to receive and use oxygen and nutrients contained in blood, as well as to communicate these metabolic demands to the cardiovascular control centre.

Maximal oxygen uptake (VO_2max_) is the international ‘gold standard’ for evaluating cardiopulmonary fitness; it measures the maximal capacity of the different aforementioned systems during a long-time intensive exercise, generally measured in the laboratory by incremental exercise tests. Nevertheless, the existing literature indicates that the relationship between VO_2max_ and mitochondrial function is complex, and studies could not demonstrate direct causality between these two physiological parameters (Lee and Zhang 2021; Holloszy and Coyle 1984), which warrants additional research to better understand their intricate interplay.

The mitochondria are the key cell organelle responsible for ATP production, in order to fuel the energy demands of the organism and muscle tissue (Attardi and Schatz 1988). Chronic exercise is one of the main factors triggering mitochondrial biogenesis, activating changes in mitochondrial content and function within the organism, especially in skeletal muscles (Hood et al. 2016; Holloszy 2011). Mitochondrial content has been shown to possibly increase by 50 to 100%, depending on the exercise programme used (Granata et al. 2018; Gordon et al. 2001).

Mitochondria, as a probable result of endosymbiotic evolution, own their own genomes. Although mitochondrial DNA (mtDNA) is quite diverse in the eukaryotic kingdom, the organisation of the mammalian mitochondrial genome is significantly conserved (Attardi and Schatz 1988; Clayton 1992). Mitochondria possess a unique genetic system, which includes multiple copies of mtDNA for each cell (Aryaman et al. 2018).

The mtDNA copy number, which is proportional to the volume of mtDNA in a cell, is frequently analysed as a measure of mitochondrial function. For example, it has been measured in the study of several diseases and conditions such as ageing, infantile neurogenetic disorders, hypotonia, developmental delays in early childhood, multiple sclerosis, renal cell carcinoma, liver disease, biliary atresia, type 2 diabetes, cardiomyopathy and breast cancer (Filograna et al. 2021). Furthermore, two studies observed a higher content of mtDNA copy number in the peripheral blood of elite athletes (swimmers and soccer players) compared to healthy controls (Galeandro et al. 2017; Baykara et al. 2016). In addition to these two studies, it has been shown that analysing mtDNA copy number in blood is biologically meaningful (Tian et al. 2021). This measure may serve as a proxy for mitochondrial function in skeletal muscle and represents a simpler, non-invasive alternative to muscle biopsies.

mtDNA copy number analysis seems to represent an innovative and potentially valuable technique to better understand the effects of aerobic performance. By directly measuring mitochondrial biogenesis, this approach offers a distinct perspective on the role of mitochondria in aerobic capacity and energy production. Furthermore, mtDNA copy number analysis may provide additional information on the molecular mechanisms underlying aerobic performance captured by VO_2max_ analysis, making it a promising avenue for future research in this field. Overall, mtDNA copy number analysis has the potential to improve our understanding of the complex relationship between mitochondria and aerobic performance, and may offer a valuable tool for researchers in the field of exercise physiology and sports science.

For these reasons, the objective of this study was first to compare VO_2max_ and mtDNA copy number in blood leukocyte between highly trained runners and nontrained male subjects, and second to study the possible adaptation of the leukocyte mtDNA content to a 6-week HIIT aerobic training intervention in the nontrained population. We hypothesised that highly trained runners would exhibit a higher mtDNA copy number than nontrained individuals, and that the mtDNA copy number would increase following the 6-week HIIT aerobic training intervention in the nontrained group.

To our knowledge, this is the first instance where an aerobic training intervention has been implemented to observe the response of mtDNA copy number to training. In addition, although it has already been studied in endurance athletes, such as swimmers or soccer players, it is the first time that the mtDNA copy number has been studied in highly trained runners.

## Methods

The study protocol adheres to the Ethics Guidelines of the Declaration of Helsinki. It was approved by the Ethics Committee of the Regional Clinical Research of the Community of Madrid (CEIm-R) approval no. Ref: 47/764390.9/17. All participants gave their written informed consent and their consent to publish.

### Design

The first part of this project was a cross-sectional study comparing a group of highly trained runners and a group of nontrained healthy subjects. For each subject, all data were collected during the same day: physical activity and cardiovascular risk stratification questionnaires, anthropometric variables, blood extraction, and an incremental treadmill test to obtain maximum heart rate (HR_max_) and VO_2max_.

The second part of the project was a randomised controlled intervention training programme; the subjects were randomly attributed to: (i) the intervention group performing a 6-week intervention of aerobic high-intensity interval training (HIIT) training programme or (ii) the control group who maintained their usual lifestyle. For each subject, all data were collected at two different times before (pre) and after (post) the 6 weeks. The Excel software random number generation function has been used to randomly assign patients to each group (1:1 allocation ratio).

### Subjects

40 subjects participated in the cross-sectional study: 20 highly trained amateur runners and 20 subjects who do not exercise regularly. Inclusion criteria for both groups were to be a man, between 20 and 45 years old, non-smoker and who obtained a “non-risk” result from the ACSM Risk Stratification Screening Questionnaire (Franklin et al. 2000). The inclusion criteria for the group of highly trained runners were: to be an amateur runner, to perform regular training at least 6 days per week, and to have a VO_2max_ above 50 ml/kg/min. The inclusion criteria for the nontrained group were to have a result inferior to 150 min of moderate physical activity per week or to 75 min of vigorous physical activity per week at the GPAQ survey of physical activity level (Armstrong and Bull 2006) and to have a VO_2max_ below 45 ml/kg/min. Runner subjects were recruited from a running club in the city of Madrid, Spain. Nontrained subjects were recruited in a fitness centre, choosing members who did not attend the centre in the previous 6 months.

For the intervention study, the same nontrained subjects were analysed again (all of them were still complying with the inclusion criteria), and 12 new subjects were recruited following the same inclusion criteria.

### HIIT aerobic training intervention

The intervention consisted of a 6-week HIIT training programme, oriented to improve aerobic capacity. The subjects were training for treadmill running 3 times per week. The first week was a familiarisation week; the first session consisted of running at 50% of their HR_max_ during 30 min, the second session at 75% of HR_max_ during 30 min, and the third session at 75% of HR_max_ during 40 min. From the second week, all the sessions started with a 5 min warm-up running at 50% of HR_max_ and ended with a 5 min cool-down at 75% of HR_max_. The second week was oriented to progressively introduce HIIT training: first session: 4 series of 2 min at 90% of HR_max_ followed by 2 min at 50% of HR_max_; second session: 4 series of 3 min at 90% of HR_max_ followed by 3 min at 50% of HR_max_. From the third session of the second week to the end of the intervention, all the sessions consisted of 4 series of 4 min at 90% of HR_max_ followed by 4 min at 50% of HR_max_.

### Anthropometric evaluation

Body composition variables were measured using the InBody 720 body composition analyser (Biospace, Seoul, Korea) after a minimum fasting period of 2 h. Weight (kg), body mass index (BMI) (kg/m^2^), body fat percentage (%), total fat (kg), and musculoskeletal mass (kg) were obtained.

### Cardiorespiratory fitness assessment

VO_2max_ was measured using a breath-by-breath gas analyser with a metabolic cart (ULTIMA series, MEDGRAPHICS, cardiorespiratory diagnostic). An incremental test was performed starting with a 3 min warm-up at 7 km/h, followed by 0.3 km/h increases of speed every 30 s, until exhaustion. The test ended when the participants reached 95% of their theoretical maximum heart rate or when their perception of exertion according to the Rated Perceived Exertion Scale (RPE) was 9/10 or “very very strong”.

### Blood sampling and DNA extraction

Blood samples were collected from the antecubital vein into a vacuum tube containing EDTA (2.5 ml) and stored at − 80 °C until analysis. To minimise potential confounding factors that may influence mtDNA levels, all participants were scheduled for evaluation in the afternoon, between 4:00 PM and 6:00 PM. They were instructed to refrain from engaging in physical exercise for at least 24 h prior to testing and to abstain from food intake for at least 3 h before the assessment.

Genomic DNA was extracted from blood samples collected with the commercial High Pure PCR Template Preparation Kit (Roche). The extracted samples were kept in the *Universidad Europea de Madrid* (UEM) Biomedicine laboratory at − 20 °C for analysis.

### Measurements of leukocyte mtDNA copy number

The mtDNA copy number was determined from extracted genomic DNA by real-time polymerase chain reaction using the PowerUp SYBR Green Master Mix (ThermoFisher) and 50 ng of genomic DNA. The threshold cycle ratio of the *mitochondria-encoded gene cytochrome c oxidase I *(*COX1*) and the *nuclear β-globin *(*HBB*) gene was analysed. The forward and reverse primers for the *nuclear β-globin *(*HBB*) gene were 5’-GAAGAGCCAAGGACAGGTAC-3’ and 5’-CAACTTCATCCACGTTCACC-3’ (Chang et al. 2016), respectively, and the forward and reverse primers for the mitochondrial gene *COX1* were: 5´-TTCGCCGACCGTTGACTATTCTCT-3´ and reverse 5´-AAGATTATTACAAATGCATGGGC-3´ (adapted from García-Merino et al. 2022), respectively. After denaturation at 95 °C for 300 s, the samples were exposed to 40 cycles of incubation at 95 °C for 0.1 s, 58 °C for 6 s, and 72 °C for 18 s. The threshold cycle number (Ct) was defined as the number of polymerase chain reaction cycles needed for the amplified DNA to reach a detectable level of fluorescence, regardless of the specific mass. Assays were performed in triplicate in triple parallel tubes; additionally, an internal control was included in each run to ensure consistency and comparability across plates. For this procedure, the intra-assay coefficient of variation (CV) had to be less than 10%. The mtDNA copy number was calculated using the following equation: relative copy number = 2_ΔCt_ (ΔCt = Ct_β-globin_ − Ct_COX1_). Melting curve analysis verified a single PCR product.

### Statistical analyses

The sample size was calculated using the G*Power 3.1.9.4 software (Faul et al. 2007) and the published data of a study by Galeandro and colleagues (Galeandro et al. 2017) with a population similar to ours. Considering the conditions of the study, we expected to achieve a 20% increase in mtDNA copy number after the intervention. Using a confidence level of 0.05 and a power 1-β of 0.8, we determined that 14 participants per group would be needed to achieve statistical significance in the main variable. All statistical analyses were performed with SPSS 25.0 (IBM Corp. Released in 2012. IBM SPSS Statistics for Windows, Version 25.0. Armonk, NY: IBM Corp.). The normality of the variables was determined using the Shapiro–Wilk test. Depending on the normality, Student’s t test for independent samples or Mann–Whitney *U* test were used to compare the means of the quantitative variables between the groups. The linear correlation between the mtDNA copy number and VO_2max_ was analysed using Pearson’s or Spearman’s correlation coefficients. Repeated measures ANOVA or Friedman’s test were used to evaluate changes over time between the HIIT and control groups before and after the 6-week intervention. Repeated measures ANOVA assumptions were assessed prior to analysis: normality of residuals was evaluated using the Shapiro–Wilk test and Q–Q plots; sphericity was tested using Mauchly’s test, and when violated, the Greenhouse–Geisser correction was applied. Independence of observations between subjects was assumed based on the study design. Post hoc comparisons between time points were adjusted using the Bonferroni correction to control for multiple testing. If needed, the mtDNA copy numbers were logarithmically transformed in order to approximate better to a normal distribution. Descriptive data were expressed as means ± standard deviation (SD), and statistical significance was assumed to be *p* < 0.05.

## Results

### Cross-sectional study

Participants from both groups presented similar results for age, height, weight and BMI (but with significant differences in body fat percentage, the subjects from the highly trained runner group being leaner than those from the nontrained group); see Table 1. Due to the inclusion criteria, the VO_2max_ results presented significant differences between the two groups.

**Table 1:** Demographic results, cross-sectional study

	**Runners** (n = 20) Mean ± SD; [95% CI]	**Nontrained** (n = 20) Mean ± SD; [95% CI]	**p**
**Age** (years)	34.5 ± 6.0; [31.7, 37.3]	37.0 ± 7.2; [33.6, 40.3]	0.248 (d = 0.37; 95% CI: [-0.26, 0.99]; small effect size)
**Height** (cm)	175.0 ± 7.2; [171.6, 178.3]	176.0 ± 7.2; [172.8, 179.6]	0.579 (d = 0.18; 95% CI: [-0.45, 0.80]; small effect size)
**Weight** (kg)	75.1 ± 6.0; [72.3, 77.9]	78.2 ± 9.1; [73.9, 82.5]	0.209 (d = 0.40; 95% CI: [-0.23, 1.03]; medium effect size)
**BMI** (kg/m^2^)	24.8 ± 1.5; [24.1, 25.5]	25.4 ± 2.2; [24.4, 26.4]	0.313 (d = 0.32; 95% CI: [-0.30, 0.95]; small effect size)
**Fat%**	18.2 ± 4.5; [16.1, 20.3]	23.0 ± 6.5; [19.8, 26.3]	0.011* (d = 0.87; 95% CI: [0.20, 1.53]; large effect size)
**VO**_**2max**_	60.5 ± 4.1; [58.6, 62.4]	39.7 ± 4.0; [37.8, 41.5]	< 0.001* (d = 5.16; 95% CI: [3.84, 6.46]; large effect size)

BMI: Body Mass Index, VO_2max_: maximum oxygen uptake, 95% CI: 95% confidence interval for the mean, * = p value < 0.05

**Fig. 1:**
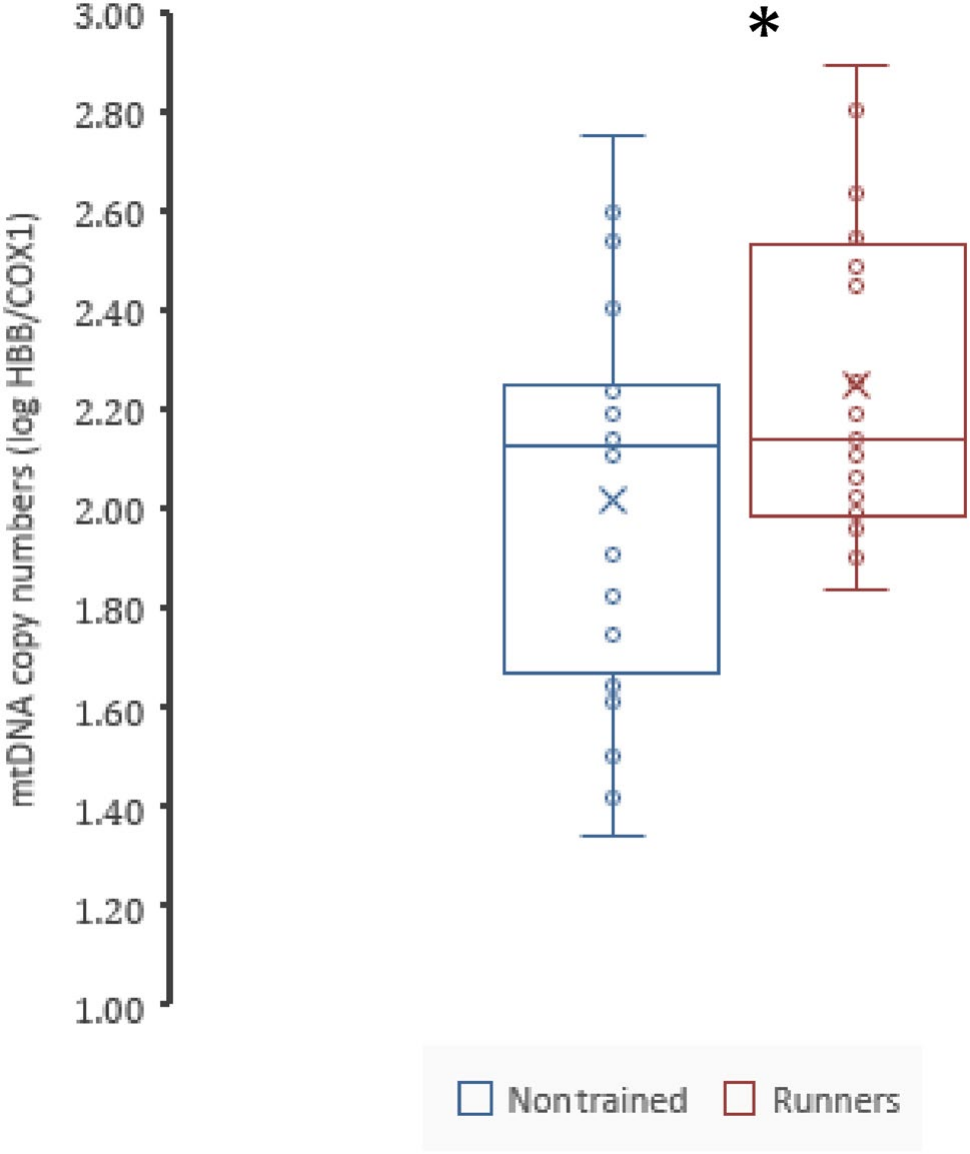
Box plot showing the comparison of the mtDNA copy number (log HBB/COX1) between the nontrained group and the runners’ group (*p* = 0.046; *d* = 0.65; 95% CI: [0.01, 1.28], [medium to large effect size])

A significant difference was found comparing the highly trained runners vs. the nontrained group for the mtDNA copy number in blood leukocytes, the trained group presenting higher values (2.25 ± 0.32; 95% CI: [2.10, 2.40] vs. 2.02 ± 0.40; 95% CI: [1.83, 2.21] log HBB/COX1; *p* = 0.046), with a Cohen’s d = 0.652; 95% CI: [0.01, 1.28] corresponding to a medium effect size. See Fig. 1.

**Fig. 2:**
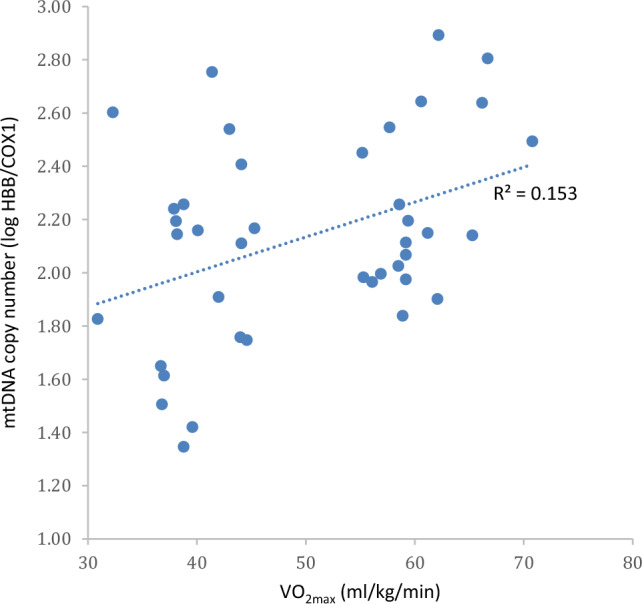
Scatter plots showing linear correlation between the mtDNA copy number (log HBB/COX1) and VO_2max_ (*p* = 0.013)

The linear correlation between mtDNA copy number and VO_2max_ combining both groups also presented significant results (*p* = 0.013) with a Pearson’s correlation coefficient *r* = 0.39; 95% CI: [0.09, 0.63] (moderate positive correlation). See Fig. 2.

### Intervention study

Participants from both groups presented similar results for age. A significant difference appears for height between the two groups, but weight was similar (pre and post intervention) and BMI was not affected by this height difference (both before and after intervention), as well as the percentage of body fat; see Table 2.

**Table 2:** Main results, intervention study

	**Intervention** (n = 17) Mean ± SD; [95% CI]	**Control** (n = 15) Mean ± SD; [95% CI]	**p**
**Age** (years)	36.9 ± 5.8; [33.4, 41.3]	35.2 ± 7.7; [29.8, 42.7]	0.489 (d = 0.25; 95% CI: [-0.45, 0.94]; small effect size)
**Height** (cm)	176.4 ± 7.0; [171.1, 179.0]	171.8 ± 4.2; [167.9, 175.2]	0.032* (d = 0.80; 95% CI: [0.07, 1.51]; large effect size)
**Weight pre** (kg)	81.5 ± 11.4; [71.4, 82.1]	75.4 ± 12.1; [62.9, 81.3]	0.148 (d = 0.53; 95% CI: [-0.19, 1.23]; medium effect size)
**Weight post** (kg)	80.7 ± 11.7; [70.1, 81.0]	75.2 ± 12.2; [62.4, 80.8]	0.211 (d = 0.45; 95% CI: [-0.26, 1.15]; medium effect size)
**BMI pre** (kg/m^2^)	26.1 ± 2.9; [23.9, 26.1]	25.5 ± 3.7; [21.8, 27.0]	0.581 (d = 0.20; 95% CI: [-0.50, 0.89]; small effect size)
**BMI post** (kg/m^2^)	25.9 ± 3.0; [23.5, 25.8]	25.4 ± 3.7; [21.7, 26.9]	0.736 (d = 0.12; 95% CI: [-0.58, 0.82]; small effect size)
**Fat% pre**	23.5 ± 6.7; [17.7, 26.3]	24.2 ± 5.7; [17.9, 26.5]	0.747 (d = -0.12; 95% CI: [-0.81, 0.58]; small effect size)
**Fat% post**	23.2 ± 6.3; [17.5, 25.0]	24.1 ± 5.9; [17.6, 26.5]	0.674 (d = -0.15; 95% CI: [-0.84, 0.55]; small effect size)
**VO**_**2max**_** pre** (ml/kg/min)	39.2 ± 3.9; [36.5, 41.9]	38.9 ± 4.5; [36.3, 44.0]	0.795 (d = 0.09; 95% CI: [-0.60, 0.79]; small effect size)
**VO**_**2max**_** post** (ml/kg/min)	44.8 ± 2.8; [42.7, 46.4]	39.6 ± 4.9; [36.3, 45.2]	< 0.001* (d = 1.33; 95% CI: [0.55, 2.09]; large effect size)
**HBB/COX1 pre**	90.3 ± 63.7; [36.4, 94.2]	103.0 ± 59.7; [49.5, 127.6]	0.552 (d = -0.21; 95% CI: [-0.91, 0.49]; small effect size)
**HBB/COX1 post**	228.7 ± 185.7; [164.7, 406.8]	113.0 ± 66.2; [67.3, 171.3]	0.029* (d = 0.81; 95% CI: [0.08, 1.53]; large effect size)

BMI: Body Mass Index, VO_2max_: maximum oxygen uptake, 95% CI: 95% confidence interval for the mean, * = p value < 0.05

At baseline, both groups presented similar results for VO_2max_. After the intervention, the intervention group presented significantly higher VO_2max_ results (*p* < 0.001; Cohen’s d = 1.33; 95% CI: [0.55, 2.09]; large effect size); see Table 2.

**Fig. 3:**
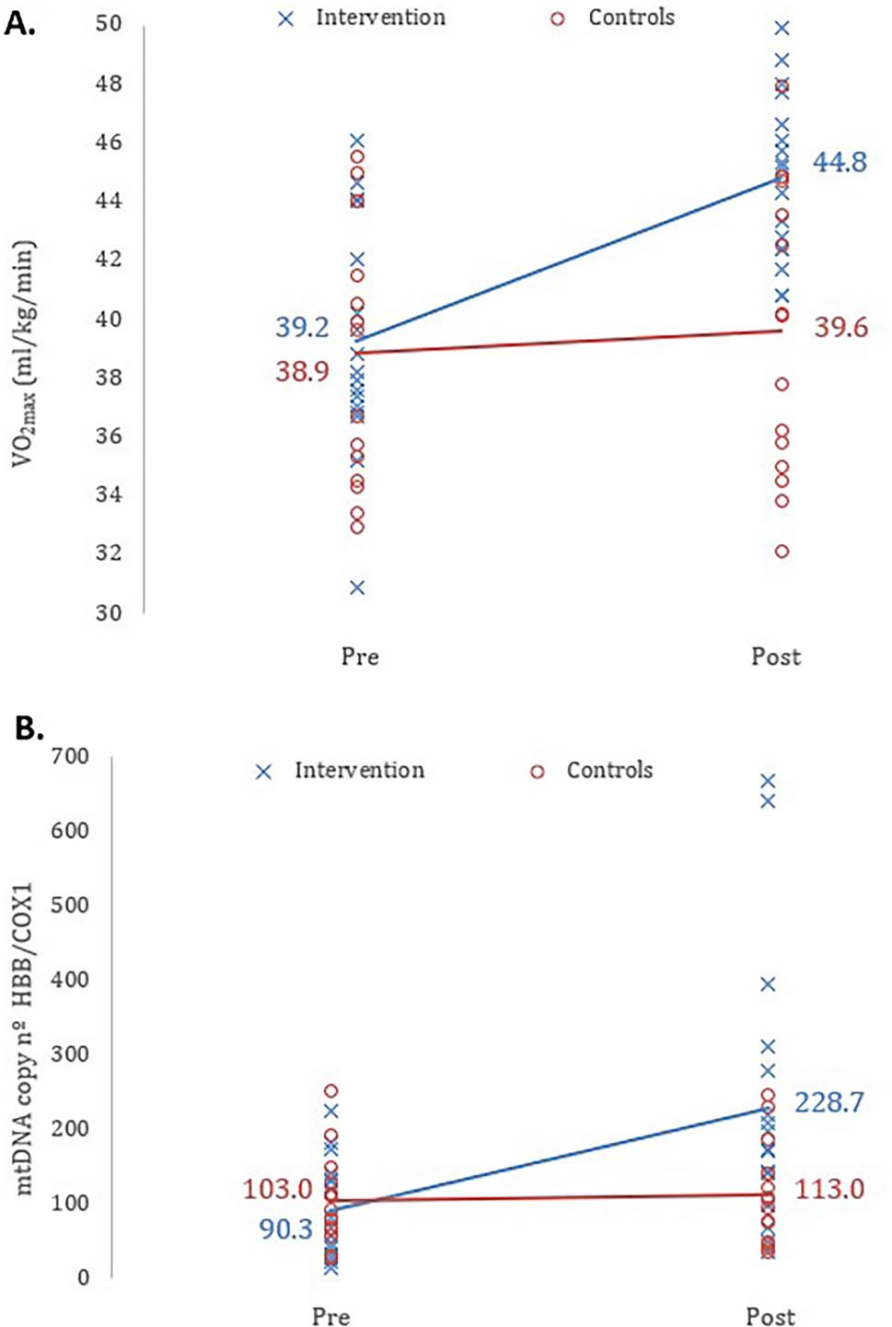
Scatter plots showing the effect of the 6-week HIIT intervention on: (**A**) the VO_2max_ (*p* < 0.001; η^2^ = 0.068 [medium effect size]) and (**B**) the mtDNA copy number (HBB/COX1 ratio) (*p* = 0.015; η^2^ = 0.072 [medium effect size]), comparing the two groups, before (Pre) and after (Post) intervention

The repeated measure analysis of variance indicated a significant time × group relation (*p* < 0.001) comparing the VO_2max_ evolution of both groups before and after intervention, with η^2^ = 0.068 indicating a medium size effect. Post hoc analysis indicated that the control group did not present significant differences in VO_2max_ before and after the intervention (*p* = 0.578); for the intervention group, VO_2max_ was significantly higher after the intervention (*p* < 0.001; η^2^_p_ = 0.121 [large effect size]) than at baseline and significantly higher than the VO_2max_ of the control group at the end of the intervention (*p* = 0.004; η^2^_p_ = 0.566 [large effect size]); see Fig. 3A.

For the analysis of the leukocytes mtDNA copy number, repeated measure analysis of variance indicated a significant time x group relation (*p* < 0.015) comparing the two groups before and after intervention, with η^2^ = 0.072 indicating a medium size effect. Post hoc analysis indicated that the control group did not present significant differences in mtDNA copy number before and after the intervention (*p* = 0.995); for the intervention group, mtDNA copy number was significantly higher after the intervention (*p* = 0.002; η^2^_p_ = 0.09 [medium-large effect size]) than at the beginning of the study. At the end of the intervention, the mean mtDNA copy number was higher in the HIIT intervention group than in the control group, but without significant differences (*p* = 0.122); see Fig. 3B. However, independent analysis of mtDNA copy number results at the end of the intervention, comparing the two groups using independent samples T-test; the HIIT intervention group presented a significantly higher mtDNA copy number than the control group, with a *p* value = 0.029 and a Cohen’s d = 0.813; 95% CI: [0.08, 1.53], corresponding to a large effect size; see Table 2. The mean percentage of increase of mtDNA copy number in the intervention group was 321.6% ± 391.6%, with a range of -165% to 1400% (95% CI: [120.2, 522.9]); in the control group, the mean increase was 12.8% ± 32.8%, with a range of -42.8% to 66.2% (95% CI: [− 5.4, 30.9]).

**Fig. 4:**
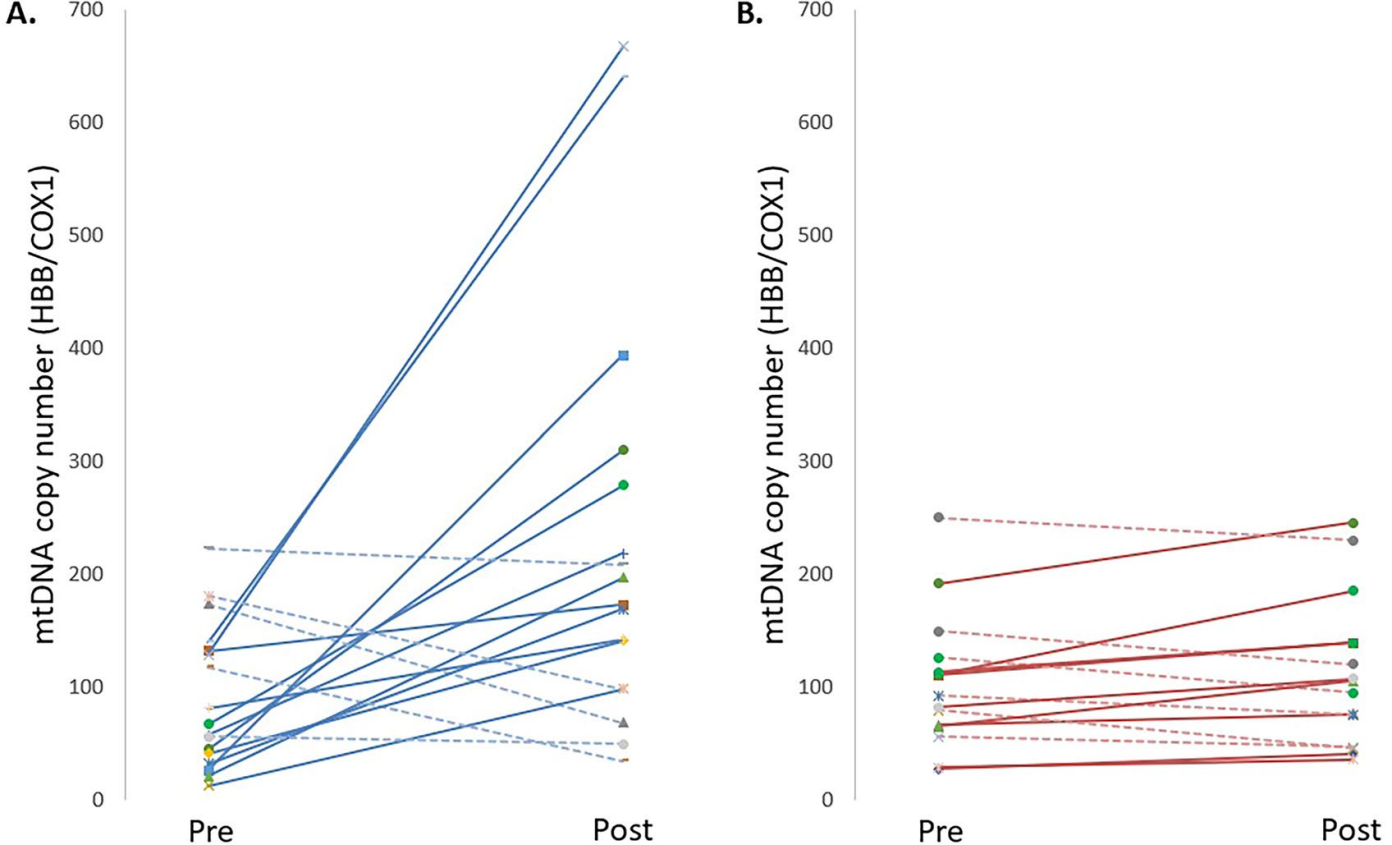
Spaghetti plots showing the evolution of the mtDNA copy number (HBB/COX1 ratio) for each subject, comparing: (**A**) the HIIT intervention group; and (**B**) the control group, before (Pre) and after (Post) 6 weeks. Continuous lines indicate subjects who increased their mtDNA copy number after 6 weeks of intervention, and dotted lines indicate subjects who decreased their mtDNA copy number. For the HIIT intervention group (**A**), a significant time × group interaction was observed (*p* < 0.001, η^2^ₚ = 0.312 [large effect size]) comparing participants who showed an increase versus those who showed a decrease

Furthermore, after 6 weeks of intervention: 12 of the 17 subjects (70.6%) in the HIIT group increased their mtDNA copy number (mean increase = 471.8 ± 372.8%; 95% CI: [234.9, 708.7]), while the other 5 subjects (29.4%) decreased their mtDNA copy number (mean decrease = 39.1 ± 28.9%; 95% CI: [3.2, 74.9]) (Fig. 4A); and 9 of the 15 subjects (60.0%) in the control group increased their mtDNA copy number (mean increase = 35.6 ± 18.0%; 95% CI: [21.8, 49.4]), while the other 6 subjects (40.0%) decreased their mtDNA copy number (mean decrease = 21.5 ± 11.8%; 95% CI: [9.2, 33.9]) (Fig. 4B).

In the HIIT group, a significant time × group interaction was observed (*p* < 0.001, η^2^ₚ = 0.312 [large effect size]) comparing participants who showed an increase (responders) versus those who showed a decrease (nonresponders). No significant differences were found between the responders and nonresponders for age or VO_2max_. The nonresponders presented significantly higher BMI, body weight and body fat percentage compared to the subjects presenting an increase in mtDNA copy number, both pre- (BMI: 27.9 ± 3.6 vs. 24.7 ± 2.5, *p* = 0.007, *d* = 1.086 (95% CI: [0.30, 1.86]); weight: 86.1 ± 12.1 vs. 74.7 ± 10.1, *p* = 0.008, *d* = 1.050 (95% CI: [0.27, 1.82]); fat%: 27.2 ± 5.0 vs. 22.1 ± 6.1, *p* = 0.025, *d* = 0.881 (95% CI: [0.11, 1.64])) and post- (BMI: 27.9 ± 3.5 vs. 24.5 ± 2.5, *p* = 0.007, *d* = 1.19 (95% CI: [0.40, 1.97]); weight: 86.2 ± 11.7 vs. 73.9 ± 10.1, *p* = 0.004, *d* = 1.16 (95% CI: [0.37, 1.94]); fat%: 27.5 ± 4.7 vs. 21.6 ± 5.8, *p* = 0.007, *d* = 1.09 (95% CI: [0.30, 1.86])) HIIT intervention.

## Discussion

In this study, in addition to a cross-sectional study comparing blood leukocytes’ mtDNA copy number in highly trained runners and in nontrained subjects, an exercise intervention was conducted for the first time that analysed the evolution of the mtDNA copy number before and after a 6-week HIIT training, in healthy nontrained subjects.

The main findings were that: (i) the highly trained runners presented significantly higher leukocytes’ mtDNA copy number than the nontrained subjects, (ii) the mtDNA copy number is significantly correlated with VO_2max_, and (iii) the 6-week HIIT intervention in nontrained subjects produced a significant increase in mtDNA copy number. Taken together, these results provide a coherent mechanistic picture: the cross-sectional data show that highly trained runners exhibit higher mtDNA copy number, while the intervention study shows that mtDNA copy number increases following a 6-week HIIT programme in previously untrained individuals. Together, these findings suggest that endurance training—whether chronic or short-term—may enhance mitochondrial biogenesis, as reflected by increased mtDNA content. This supports the idea that both long-term adaptation and short-term intervention can modulate mitochondrial content in blood leukocytes, potentially contributing to improved aerobic capacity.

The results obtained in the cross-sectional study are consistent with the previous literature: in 2016, Chang et al*.* (2016) observed a higher mtDNA copy number of peripheral leukocytes in postmenopausal women who regularly exercise than in those who do not exercise. In 2021, Hagman et al*.* (2021) found similar results in young women; a group of elite football players showed a 19–20% higher mtDNA copy number compared to an untrained control group of young women. However, they also observed that mtDNA copy number results could be affected by age: elderly women presenting a higher mtDNA copy number than young women; and in contrast, the trained elderly women showed a lower mtDNA copy number than the untrained ones. Our results comparing a group of highly trained male runners with an untrained group of men confirm previous results obtained in other sport specialities: Baykara et al*.* (2016) observed mtDNA copy number increased in relation to the level of different groups of swimmers; and Galeandro et al*.* (2017) observed a higher mtDNA copy number in professional soccer players than in healthy nonathletic subjects. However, Piotrowska-Nowak et al*.* recently obtained opposite results, observing a lower mtDNA copy number in both power and endurance male athletes compared to controls (Piotrowska-Nowak et al. 2023), emphasising a need for a more comprehensive analysis of the involvement of the mitochondrial genome in physical performance. In this sense, it is worth noting that the *p* value obtained for these results (*p* = 0.046) lies very close to the conventional threshold for statistical significance (*α* = 0.05), suggesting that the evidence against the null hypothesis is marginal and should be interpreted with caution.

The correlation observed in the first part of the present study between the mtDNA copy number and VO_2max_ is consistent with the work of Chen et al*.* (2017) observing that a greater mtDNA copy number was associated with better exercise ventilatory efficiency and higher VO_2max_ in healthy subjects.

The novel part of this project is the analysis of the 6-week long HIIT intervention. The results obtained showed that the number of copies of mtDNA is related to endurance training, as the intervention group showed a significant increase of 316 ± 399%, versus 13 ± 33% for the control group, which corresponds to the comparable results of VO_2max_ obtained in both groups (Fig. 3). It is well known that the mtDNA copy number reflects the abundance of mitochondria in cells, which can change under different energy requirements and different physiological and environmental conditions (Lee and Wei 2005). These results confirm those of previous literature mentioned above (Galeandro et al. 2017; Baykara et al. 2016; Chang et al. 2016; Hagman et al. 2021; Chen et al. 2017), stating that the increase in mtDNA copy number could be associated with sports load. This observation supports a mechanism by which performance is modulated by the ability to efficiently activate mitochondrial biogenesis in response to the high demand for aerobic ATP production. Mitochondrial biogenesis is the process of generating new mitochondria within cells, which involves continuous dynamic processes of fission and fusion cycles, which, accompanied by mitophagy and transport, determine the morphology, quality, quantity and distribution of mitochondria within cells, as well as the mitochondrial function (reviewed in Chen et al. (2023)). This dynamic process of biogenesis plays an important role in cellular adaptation to exercise: the main regulators of mitochondrial biogenesis are transcription factors, nuclear respiratory factors 1 and 2 (NRF-1 and NRF-2), the mitochondrial transcription factor A (TFAM), the p53, and the transcriptional coactivator peroxisome proliferator-activated receptor-γ coactivator-1α (PGC-1α) (reviewed in Abrego-Guandique et al. (2025)). The latter is present in tissues with a high capacity of mitochondrial systems. The expression of the PGC-1α gene is rapidly induced by exposure to cold, acute exercise, and fasting. The PGC-1α signalling cascade of mitochondrial biogenesis ending with TFAM activation plays a significant regulatory role in mtDNA transcription by binding upstream of promoters for transcription initiation and directly influencing secondary transcription factors involved in mitochondrial translation and metabolism and in regulating mtDNA transcription (reviewed in Yang et al. (2025)). However, the fact that the mtDNA is the template for proteins that participate in energy production does not mean that mtDNA copy number directly reflects mitochondrial function or energy production capacity. There are at least three intermediate steps between mtDNA copy number and energy production capacity: first, mtDNA must be transcribed into intermediate RNAs. Second, the messenger RNAs must be translated into 13 functional subunit proteins of the mitochondrial respiratory chain. Third, mtDNA-encoded proteins must assemble, along with proteins encoded in the nucleus, into functional RC complexes (I, III, IV, V): large multi-protein complexes that transfer electrons and pump protons. In total, the mitochondrion is made of > 1,300 proteins, only 13 of which are encoded in mtDNA (reviewed in Picard (2021)).

Furthermore, we also found that some subjects were nonresponders to HIIT aerobic training; 29.4% of the subjects showed a decrease in their mtDNA copy number at the end of the 6 weeks (see Fig. 4A). This high variability can be attributed to a combination of biological factors. The mtDNA copy number varies significantly across physiological states (Pastukh et al. 2016), cell cycle phases (Potapenko et al. 2024) or circadian rhythm (Wang et al. 2020). In blood tissue, mtDNA copy number also varies widely between leukocyte types (ranges from ~ 150 to 600 copies) (Picard 2021). In addition, factors such as oxidative stress (Ikeda et al. 2015) or early life stress (Beck et al. 2025) can induce changes in mitochondrial biogenesis, contributing to the observed heterogeneity. Interestingly, we observed that the nonresponders presented significantly higher BMI, body weight, and body fat percentage compared to the responders; the mean BMI of the nonresponders was above 25 kg/m^2^, corresponding to overweight (Report of a WHO Expert Committee 1995). Thus, it is well known that overweight and high body fat percentage are related to downregulation of mitochondrial biogenesis and to mitochondrial dysfunction (reviewed in Mello et al. (2018)), which could explain the results obtained.

As stated by Larsen et al*.* (2012), mtDNA content is often considered an inconsistent biomarker of mitochondrial content due to the fact that mitochondrial DNA copy number can vary considerably between individuals. Furthermore, in a review on this topic, Granata et al*.* (2018) raised the possibility that exercise-induced changes in markers of mitochondrial biogenesis may not always predict or relate to training-induced adaptations. Based on the findings of this study, leukocyte mtDNA copy number can serve as an individual follow-up biomarker during exercise interventions, particularly those that stimulate the mitochondrial biogenesis pathway. This method could be proposed as a simple, non-invasive technique to complement the analysis of cardiorespiratory capacity and mitochondrial function in relation to exercise.

Based on our findings and the existing literature, it is now apparent that the analysis of the mtDNA copy number can constitute a novel variable to consider when assessing physical condition. This measure provides a valuable complement to traditional cardiopulmonary exercise/VO_2max_ tests by offering insights more directly related to mitochondrial function. Furthermore, since our study indicates that the response of the mtDNA copy number to exercise training can be altered in sedentary individuals with a higher BMI and a higher percentage of body fat, it appears to be a significant variable to monitor during physical condition interventions in overweight or obese individuals. On the other hand, an increasing number of studies associated mitochondrial dysfunction with increased mtDNA vulnerability, mainly due to oxidative stress. This mitochondrial dysfunction has been linked to serious diseases such as Alzheimer’s or Parkinson’s (Coskun et al. 2012), various types of cancer (Mi et al. 2015), type 2 diabetes (Pinti et al. 2019), or diabetic kidney disease (Yu et al. 2024), among others. Studying the mtDNA integrity through the number of copies in healthy active and sedentary individuals and understanding its variation based on exercise intervention is useful for understanding the mechanisms that may be involved in such interventions. This way, we can understand the benefits of exercise in the context of pathology, not only at the physiological level but also at the cellular and molecular levels, and demonstrate the value of ‘mitochondrial health’ in the prevention and prognosis of certain diseases.

Regarding the interpretation of our findings, it is important to consider the methodological limitations associated with the quantification of mtDNA copy number. Although this marker has been proposed as a non-invasive proxy for mitochondrial biogenesis, its value as a functional indicator remains debated. In our study, standardised procedures were applied to minimise technical variability: mtDNA levels were normalised against nuclear DNA using the β-globin gene as a reference, following protocols validated in previous studies (Agius et al. 2022; Wu et al. 2022; Li et al. 2021). All samples were analysed in triplicate under homogeneous conditions of collection and processing, resulting in an intra-assay coefficient of variation below 10%, indicative of high reproducibility. We also acknowledge that mtDNA copy number may be influenced by physiological, environmental, and circadian factors, as well as by leukocyte subtype heterogeneity, which introduces biological variability that must be considered when interpreting the results. Despite these limitations, our findings are consistent with previous studies reporting increases in mtDNA copy number following exercise interventions, supporting its utility as a complementary marker in the study of mitochondrial adaptations to training.

This study has several limitations that should be acknowledged. First, for the intervention study, participant allocation was influenced by their scheduling availability, which introduced a degree of selection bias and limited the rigour of the randomisation process. Second, the sample size may be considered modest, although all groups in the present study exceeded the threshold determined by the a priori power analysis, supporting the statistical validity of the findings. Third, mtDNA copy number was assessed in leukocytes rather than in skeletal muscle tissue. While leukocyte mtDNA has been proposed as a non-invasive proxy for systemic mitochondrial adaptations, it may not fully capture tissue-specific mitochondrial responses to exercise, particularly in metabolically active tissues such as skeletal muscle. Moreover, leukocyte mtDNA levels can be influenced by factors such as immune status, circadian rhythms, and the relative composition of leukocyte subtypes, all of which may introduce biological variability and limit the specificity of this marker as a proxy for mitochondrial adaptations. Fourth, no direct functional assessments of mitochondrial oxidative capacity (e.g. transmission electron microscopy, high-resolution respirometry, or enzymatic activity assays) were performed. As such, the observed changes in mtDNA copy number cannot be directly linked to mitochondrial function.

In conclusion, this study confirms the results obtained in the previous literature in different sports specialties, showing that the mtDNA copy number is related to the level of aerobic capacities. We observed that a group of highly trained runners had significantly higher leukocyte mtDNA copy number than a group of untrained subjects, and that their mtDNA copy number was significantly correlated with VO_2max_. The novelty of this study was then to study the adaptations of the leukocyte mtDNA content to a 6-week HIIT aerobic training intervention in a group of untrained men. This intervention allows us to better understand the different individual adaptations to endurance training: we observed that the 6-week HIIT intervention produced a significant increase in mtDNA copy number in this group.

## Data Availability

Available on request.

## Abbreviations

ANOVA: Analysis of variance
ATP: Adenosine triphosphate
BMI: Body mass index
CEIm-R: Ethics committee of the regional clinical research of the community of Madrid
COX1: Cytochrome c oxidase I
Ct: Threshold cycle number
DNA: Deoxyribonucleic acid
HBB: Nuclear β-globin haemoglobin subunit beta
HIIT: High-intensity interval training
HR_max_: Maximum heart rate
mtDNA: Mitochondrial DNA
RPE: Rated perceived exertion scale
UEM: *Universidad Europea de Madrid* (European University of Madrid)
VO_2max_: Maximal oxygen uptake
