# Correction: Adaptation of mtDNA content to endurance training, a cross-sectional study and an endurance training intervention

**DOI:** 10.1007/s00421-026-06127-7

**Published:** 2026-03-06

**Authors:** Isabel María Sánchez Lorente, Thomas Yvert, Tamara Iturriaga, Lara Sanchez-Barroso, Mar Larrosa, Margarita Pérez-Ruiz, Catalina Santiago-Dorrego

**Affiliations:** 1 ESBIDA Research Group, Department of Sport Sciences, Faculty of Medicine, Health and Sports, Universidad Europea de Madrid, Madrid, Spain; 2 ImFINE Research Group, Department of Health and Human Performance, Universidad Politécnica de Madrid, Madrid, Spain; 3 Department of Nursing, Facultad de Enfermería, Fisioterapia y Podología, Universidad Complutense de Madrid, Madrid, Spain; 4 InveCuid, Instituto de Investigación Sanitaria Hospital 12 de Octubre (imas12), Madrid, Spain; 5 Department of Nutrition and Food Science, Faculty of Pharmacy, Universidad Complutense de Madrid, Madrid, Spain; 6 ESBIDA Research Group, Department of Rehabilitation, Faculty of Medicine, Health and Sports, Universidad Europea de Madrid, Madrid, Spain

**Correction: European Journal of Applied Physiology**

10.1007/s00421-025-06016-5

In the original version of this article, the image for Figure 4 is currently showing the second part of Figure 3, and the actual image for Figure 4 does not appear.

The Figs. 3 and 4 should have appeared as shown below.

**Fig. 3:**
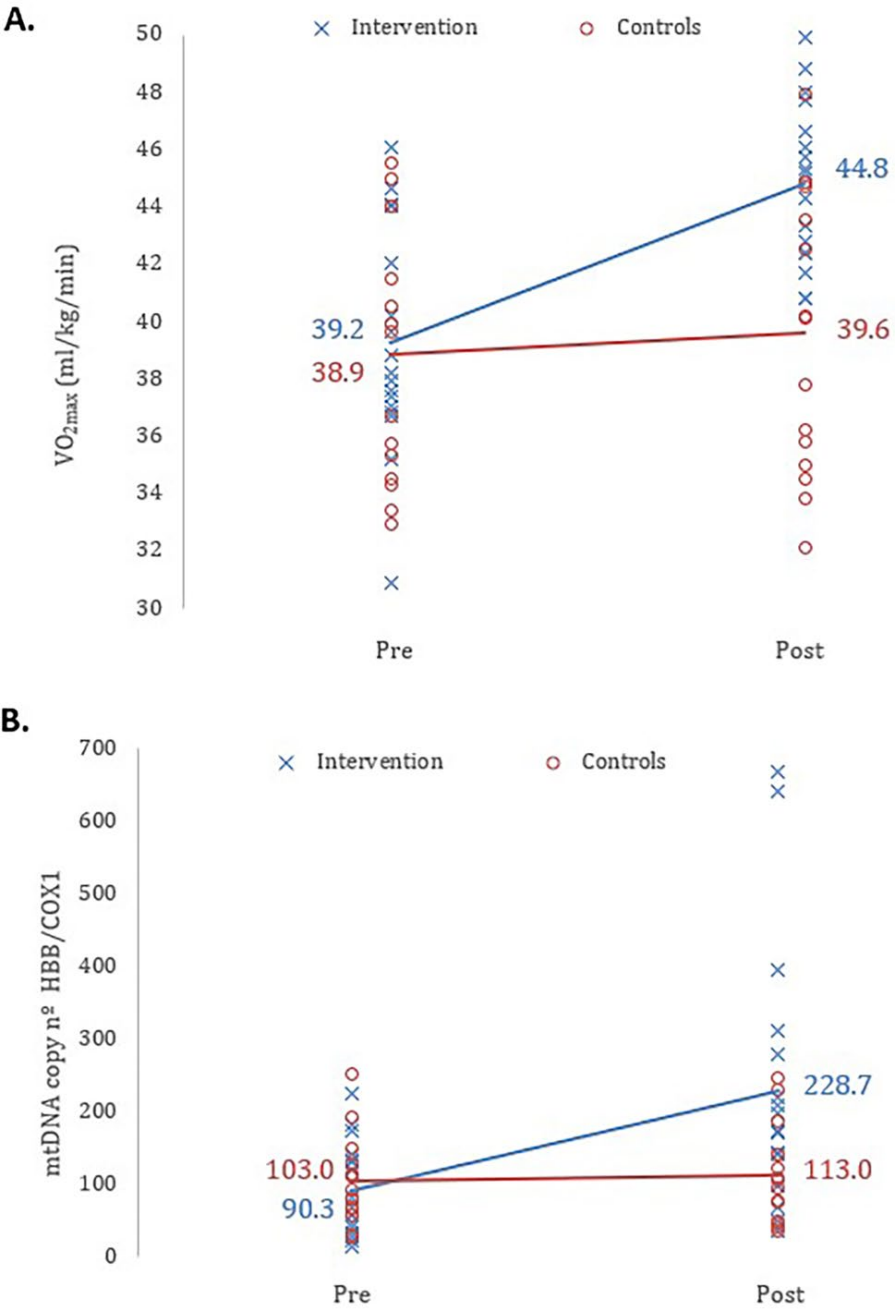
Scatter plots showing the effect of the 6 weeks HIIT intervention on: (**A**) the VO_2max_ (*p* < 0.001; η^2^ = 0.068 [medium effect size]) and (**B**) the mtDNA copy number (HBB/COX1 ratio) (*p* = 0.015; η^2^ = 0.072 [medium effect size]), comparing the two groups, before (Pre) and after (Post) intervention

**Fig. 4:**
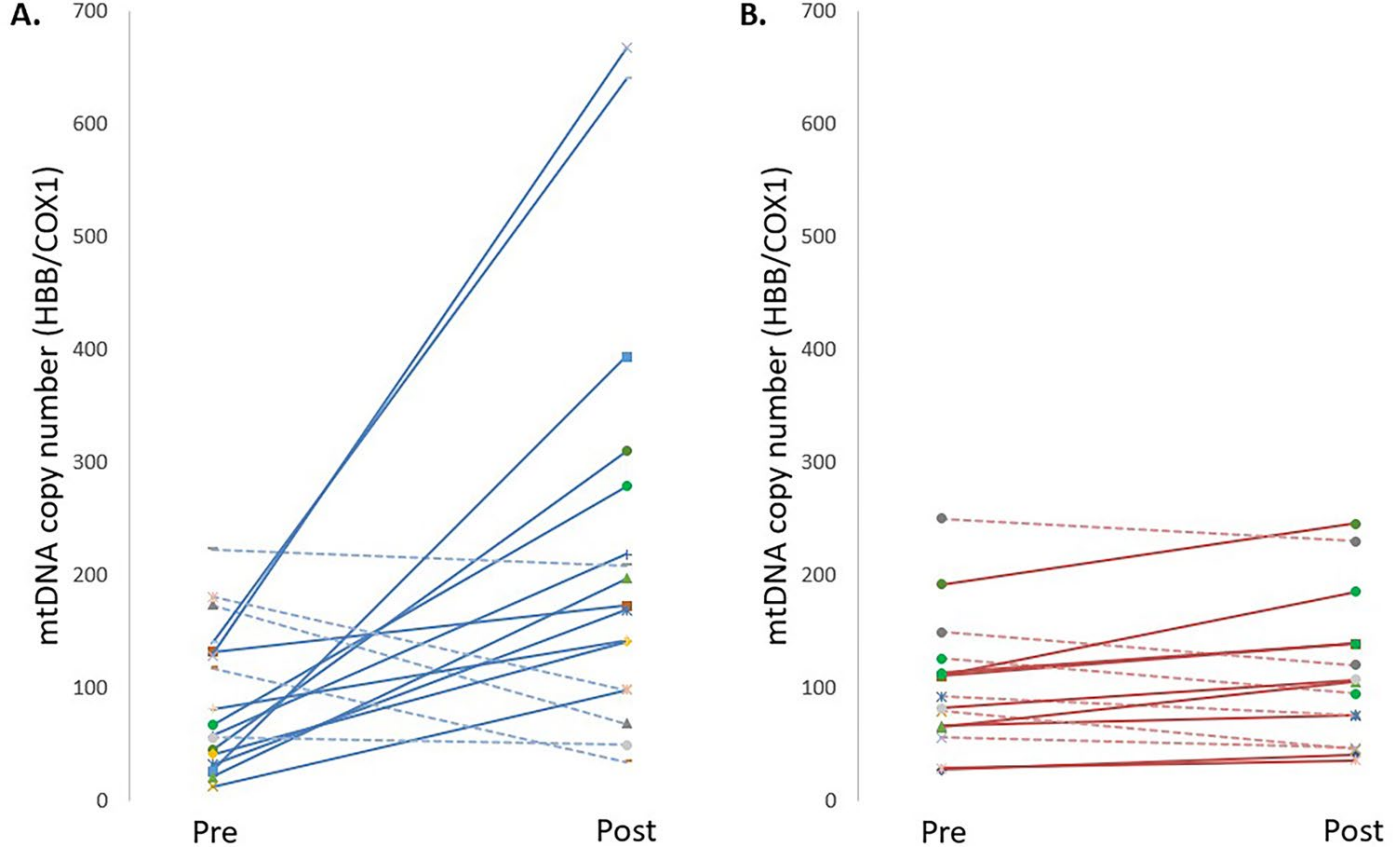
Spaghetti plots showing the evolution of the mtDNA copy number (HBB/COX1 ratio) for each subject, comparing: (**A**) the HIIT intervention group; and (**B**) the control group, before (Pre) and after (Post) 6 weeks. Continuous lines indicate subjects who increased their mtDNA copy number after 6 weeks of intervention, and dotted lines indicate subjects who decreased their mtDNA copy number. For the HIIT intervention group (**A**), a significant time × group interaction was observed (*p* < 0.001, η^2^_p_ = 0.312 [large effect size]) comparing participants who showed an increase versus those who showed a decrease

The original article has been corrected.

